# Assessing, treating and preventing community acquired pneumonia in older adults: findings from a community-wide survey of emergency room and family physicians

**DOI:** 10.1186/1471-2296-6-32

**Published:** 2005-08-02

**Authors:** Paul Krueger, Mark Loeb, Caralyn Kelly, H Gayle Edward

**Affiliations:** 1 St. Joseph's Health System Research Network, Father Sean O'Sullivan Research Centre, Hamilton, Ontario, Canada; 2 Department of Clinical Epidemiology and Biostatistics, McMaster University, Hamilton, Ontario, Canada; 3 St. Joseph's Lifecare Centre, Brantford, Ontario, Canada; 4 Department of Pathology and Molecular Medicine, McMaster University, Hamilton, Ontario, Canada; 5 Department of Family Relations and Applied Nutrition, University of Guelph, Guelph, Ontario, Canada

## Abstract

**Background:**

Respiratory infections, like pneumonia, represent an important threat to the health of older Canadians. Our objective was to determine, at a community level, family and emergency room physicians' knowledge and beliefs about community acquired pneumonia (CAP) in older adults and to describe their self-reported assessment, management and prevention strategies.

**Methods:**

All active ER and family physicians in Brant County received a mailed questionnaire. An advance notification letter and three follow-up mailings were used to maximize physician participation rate. The questionnaire collected information about physicians' assessment, management, and prevention strategies for CAP in older adults (≥60 years of age) plus demographic, training, and practice characteristics. The analysis highlights differences in approaches between office-based and emergency department physicians.

**Results:**

Seventy-seven percent of physicians completed and returned the survey. Although only 16% of physicians were very confident in assessing CAP in older adults, more than half reported CAP to be a very important health concern in their practices. In-service training for family physicians was associated with increased confidence in CAP assessment and more frequent use of diagnostic tests. Family physicians who reported always requesting chest x-rays were also more likely to request pulse oximetry (OR 5.6, 95% CI 1.40 to 22.5) and recommend both follow-up x-rays (OR 5.4, 95% CI 1.7 to 16.6) and pneumococcal vaccination (OR 3.4, 95% CI 1.1 to 10.0).

**Conclusion:**

The findings of this study provide a snapshot of how non-specialists from a non-urban Ontario community assess, manage and prevent CAP in older adults and highlight differences between office-based and emergency department physicians. This information can guide researchers and clinicians in their efforts to improve the management and prevention of CAP in older adults.

## Background

Community-acquired pneumonia (CAP) is an important threat to older adults. The majority of excess deaths and hospitalizations due to CAP occur in older adults, as reflected by over 44,000 hospitalizations for pneumonia and influenza annually in people aged 65 and older in Canada. The incidence of pneumonia increases dramatically in the very old, with rates increasing from 15 cases per 1,000 in those aged 60–74 years to 34 cases per 1,000 for those 75 years and older [1]. Clinical management decisions for CAP, such as timing of antimicrobials or site of care, can impact mortality and cost, particularly in the elderly [2,3]. Few studies, however, have examined provision of care for CAP in entire communities. A vaccination trial set in a township in Finland allowed researchers the opportunity to assess etiology, incidence, and risk factors for CAP [4-6]. However, health service delivery for CAP was not addressed. In this paper, we report the findings from a community-wide survey of family and emergency room (ER) physicians from one Ontario community to assess knowledge and beliefs about CAP and ascertain variation in management strategies.

## Methods

### Setting

This study was conducted in Brant County, which includes the city of Brantford and the amalgamated County of Brant. The population of Brant County in 2001 was 118,485, with 14% of the population aged 65 years and older [7]. There are two community hospitals, eight radiology centres, and approximately 80 family physicians who serve older adults in Brant County. Brant County was selected for this community-wide study because of its moderate size and population demographics. It is a predominantly English speaking community with 86% of the population reporting English as a first language.

### Selection of physicians

Patients presenting with CAP in Brant County are first assessed either in a primary care office setting or in the hospital emergency department. Therefore, the target population for our survey included family and ER physicians. We created a comprehensive up-to-date list of all family and ER physicians practising in Brant County using several sources: hospital physician lists; the local telephone book; and The College of Physicians and Surgeons of Ontario's Doctor Search Internet database [8]. This list was then reviewed by research team members, two local family physicians, a hospital administrator and selected hospital staff to ensure accuracy and completeness.

### Questionnaire

Using a framework described by Dillman, a questionnaire was developed to collect information about the assessment, management and prevention of CAP in older adults [9]. The questionnaire was pretested by two family physicians prior to implementation. An advance notification letter and three follow-up mailings were used to help maximize the response rate. An advance notification letter informed physicians of the survey's purpose and assured respondents of complete confidentiality. One week later the questionnaire, a return postage-paid envelope and a cover letter explaining the study's purpose was mailed to all physicians. An incentive (booklet of five $1 coupons for coffee) was also included in the package as a token of appreciation. Physicians were notified that they could opt out of receiving follow-up mailings by returning their blank questionnaire with a note stating they were not interested in participating. The following week a thank you/reminder letter was mailed to all physicians. Two weeks later a second package (without the incentive) was mailed to all physicians who had not yet responded. Two weeks later, a final package was sent by registered mail to non-responding physicians. This research received ethics approval from McMaster University.

### Statistical analysis

Data from the mailed questionnaires were entered into and analyzed using SPSS 12.0.0 (SPSS, Inc., Chicago IL). Descriptive statistics were computed for all variables measured, including frequency counts and percentages, or means and standard deviations (as appropriate). We used the chi-square test or, when appropriate, Fisher's exact test to determine differences in categorical variables. Unadjusted odds ratios (ORs), 95% confidence intervals (CIs), and p-values are reported as appropriate. A probability level of <0.05 was used to determine statistical significance.

## Results

### Response rate

Of 98 eligible physicians, 75 (77%) returned completed questionnaires. The response rate varied by type of physician with 78% (63/81) of family physicians and 71% (12/17) of ER physicians responding.

### Respondent characteristics

Of the 75 respondents, 28 (37%) classified their type of practice as "solo practice", 19 (25%) as "family physician group practice (with other family physicians)", 12 (16%) as ER, 6 (8%) as "family physician/specialist group practice (with other physician/dental specialists)", 5 (7%) as "acute & urgent care", 1 (1%) as "multi-disciplinary group practice (with independent practitioners other than MDs)", and 4 as "other" (2 walk-in clinic, 1 locum, and 1 hospitalist). Fifty-nine (79%) of the physicians reported their method of reimbursement as fee for service; 11 (15%) as salary (hospitalist and ER physicians); 3 (4%) as capitation and 2 (3%) as sessional payment (ER physicians). Seventy-two percent of respondents were male and length of time in practice ranged from one to 51 years (with a mean of 22 years). Forty-one percent of respondents reported having an undergraduate degree (other than medicine); 60% a CCFP; and 10% a graduate degree (e.g. MSc, MA).

### Attitudes and knowledge about CAP

When asked to rate the importance of CAP as a health concern for older adults in their practices, 51% of physicians reported it to be very important. Although a higher percentage of ER physicians than family physicians reported CAP to be very important (58% vs 49% respectively) this difference was not statistically significant (p = 0.56). An additional 43% rated CAP as being fairly important in their practices. Only 16% of respondents reported being very confident in assessing CAP in older adults. ER physicians were significantly more likely (p = 0.02) than family physicians to respond that they were very confident in their assessments, 42% vs 11% respectively (OR 5.7, 95% CI 1.4 to 22.9, p = 0.019). An additional 75% reported being fairly confident in such assessments. Regarding education about CAP, 77% of respondents reported having attended continuing medical education (CME) events with a focus on CAP in older adults. In addition to attending CME events, respondents reported obtaining information related to assessing and treating CAP in older adults from a number of sources including: journal articles (92%), discussions with colleagues (79%), pharmaceutical representatives (55%), in-service training (12%) and the Internet (5%). Family physicians who received in-service training related to assessing and treating CAP in older adults were significantly more likely (p = 0.03) than those who did not receive such training to report being very confident in assessing CAP in older adults (OR 13.6, 95% CI 2.4 to 78.8).

Only 39% of respondents agreed or strongly agreed that their undergraduate medical education provided adequate training in assessing and treating CAP in older adults. This increased to 72% when asked about their postgraduate medical education.

### Assessment and diagnostic testing

The questionnaire included an extensive list of 24 signs and symptoms that have been associated with CAP in older adults. Physicians were asked to report how frequently (always, usually, occasionally, rarely, never) they see these signs and symptoms in older adults clinically diagnosed with CAP. The most common symptoms or signs, always or usually reported, were fatigue (84%), abnormal breath sounds (81%), shortness of breath (72%), and productive cough (64%). The questionnaire also provided physicians with a list of patient characteristics and asked them to rate how important this information was when assessing and treating older adults suspected of having CAP. Characteristics that were reported as being very important to know included: other co-morbidities (73%), smoking status (61%), hydration (57%), social support (32%), age (25%), cognitive impairment (20%), alcohol consumption (19%), caregiver burden (17%), and physical disabilities (13%). ER physicians were significantly more likely (p = 0.04) to report age as being very important to know compared to family physicians (50% vs 21% respectively).

Physicians were also asked to indicate, from a list of tests, how often (always, usually, occasionally, rarely, never) they normally request each test when they suspect an older adult has CAP (Table 1). Although over 90% of family and ER physicians always or usually ordered chest radiographs, ER physicians were significantly more likely (p = 0.01) than family physicians to always request chest x-rays (92% vs 51% respectively). ER physicians were also more likely to always or usually order complete blood count (92%) and pulse oximetry (100%) than family physicians (52% and 24% respectively).

**Table 1:** Frequency that tests are normally requested when older adults are suspected of having CAP.

	**Family Physicians**			**ER Physicians**			
		
**Test**	**Always %**	**Usually %**	***Combined %***	**Always %**	**Usually %**	***Combined %***	P-value*
Chest X-ray	50.8	41.3	*92.1*	91.7	8.3	*100*	>0.05
CBC	14.3	38.1	*52.4*	58.3	33.3	*91.6*	<0.05
Pulse oximetry	14.3	9.5	*23.9*	91.7	8.3	*100*	<0.001
Sputum culture	1.6	11.3	*16.1*	0.0	33.3	*33.3*	>0.05
Blood culture	3.2	6.5	*9.7*	16.7	50.0	*66.7*	<0.001
Sputum gram stain	1.6	3.2	*4.8*	0.0	25.0	*25.0*	<0.05
Arterial blood gas	1.6	1.6	*3.2*	0.0	8.3	*8.3*	>0.05

*Comparison of "combined" percentages.

Family physicians who reported always requesting chest x-rays when older adults were suspected of having CAP were significantly more likely than those who did not always request chest x-rays to also report: usually or always requesting pulse oximetry (OR 5.60, 95% CI 1.4 to 22.5, p = 0.010); always requesting follow-up x-rays (OR 5.4, 95% CI 1.7 to 16.6, p = 0.003); and always recommending pneumococcal vaccine (OR 3.4, 95% CI 1.1 to 10.0, p = 0.027). Family physicians who reported usually or always requesting pulse oximetry when older adults were suspected of having CAP were significantly more likely than those who did not to also report: CAP as being a very important concern for older adults in their practice (OR 3.9, 95% CI 1.1 to 13.9, p = 0.032); always requesting chest x-rays when older adults are suspected of having CAP (OR 5.6, 95% CI 1.4 to 22.5, p = 0.010); usually or always using the Pneumonia Severity Index (PSI) to assist clinical judgement in admitting an older adult to hospital (OR 11.7, 95% CI 2.0 to 69.9, p = 0.007; obtaining information related to assessing and treating CAP in older adults via in-service training (OR 5.5, 95% CI 1.3 to 24.2, p = 0.029); and not having their primary method of reimbursement as fee-for-service (OR 11.9, 95% CI 1.1 to 125.0, p = 0.039).

### Therapeutic management and site of care

Physicians were asked which antibiotics they normally prescribe for treating outpatient, immunocompetent, older adults with CAP. They were asked to list their first and second choices along with treatment duration. The most common antimicrobials reported by 57 family physicians were newer macrolides (by 43 or 75%), respiratory fluoroquinolones (by 6 or 11%), and beta-lactams (by 4 or 7%). The most common antimicrobials reported by 12 emergency department physicians were respiratory fluoroquinolones (by 6 or 50%), newer macrolides (by 3 or 25%), and beta-lactams (by 3 or 25%). There was very little variation in treatment duration by either ER or family physicians. Most reported prescribing these antimicrobials for seven to 10 days.

Ancillary therapy and follow-up strategies are summarized in Table 2. Use of analgesics and follow-up chest radiograph were commonly practised by the physicians surveyed. Family physicians who reported always requesting follow-up chest x-rays for older patients clinically diagnosed with CAP were significantly more likely than those who did not to: also report CAP as being a very important concern for older adults in their practice (OR 4.3, 95% CI 1.5 to 13.0, p = 0.007); and always requesting chest x-rays when older adults are suspected of having CAP (OR 5.4, 95% CI 1.7 to 16.6, p = 0.003).

**Table 2:** Frequency that physicians normally prescribe/recommend management strategies for older adults with clinically diagnosed CAP.

	**Family Physicians**			**ER Physicians**			
		
**Management Strategy**	**Always %**	**Usually %**	***Combined %***	**Always %**	**Usually %**	***Combined %***	P-value*
Analgesics / antipyretics	14.5	66.1	*80.6*	8.3	83.3	*91.6*	>0.05
Follow-up chest x-ray	38.1	39.7	*77.8*	8.3	50.0	*58.3*	>0.05
Follow-up appointment (24–48 hrs)	21.0	41.9	*62.9*	33.3	58.3	*91.6*	>0.05
Hydration	19.4	40.3	*59.7*	25.0	41.7	*66.7*	>0.05
Respiratory therapy	1.6	9.7	*11.3*	8.3	25.0	*33.3*	>0.05
Oxygen therapy	1.6	4.8	*6.4*	8.3	41.7	*50.0*	<0.001
Referral to ER	1.6	3.2	*4.8*	n/a	n/a	*n/a*	-
Referral to physician specialist	0.0	4.8	*4.8*	0.00	16.7	*16.7*	>0.05
Hospital admission	0.0	3.3	*3.3*	0.0	25.0	*25.0*	<0.05
Home care services							
Nursing care	0.0	10.0	*10.0*	0.0	10.0	*10.0*	>0.05
Nutrition assessment	0.0	3.4	*3.4*	0.0	10.0	*10.0*	>0.05
Homemaking	0.0	6.7	*6.7*	0.0	10.0	*10.0*	>0.05
IV antibiotic therapy	0.0	3.4	*3.4*	0.0	9.1	*9.1*	>0.05

*Comparison of "combined" percentages.

Physicians were asked how often they used the Pneumonia Severity Index (or similar decision tools) to assist their clinical judgement in admitting an older adult to hospital. Most family physicians (67%) reported rarely or never while half (50%) of ER physicians reported usually or always using the PSI. Some of the family physicians commented that they had no hospital privileges and others that they had never seen the PSI. Family physicians who reported usually or always using the PSI to assist clinical judgement in admitting an older adult to hospital were significantly more likely than those who did not to also report: obtaining information related to assessing and treating CAP in older adults via in-service training (OR 12.3, 95% CI 2.1 to 71.2, p = 0.008); and usually or always requesting pulse oximetry (OR 11.7, 95% CI 2.0 to 69.9, p = 0.007).

### Preventing CAP in older adults

Physicians were asked how frequently (always, usually, occasionally, rarely, never) they recommended various prevention strategies to older adults in their practice setting (Table 3). Family physicians who reported always recommending annual influenza vaccine to older patients were significantly more likely than those who did not to also report always recommending pneumococcal vaccine to older patients (OR 1.7, 95% CI 1.2 to 2.4, p < 0.001). Family physicians who reported always recommending pneumococcal vaccine to older patients were significantly more likely than those who did not to also report: always requesting chest x-rays when older adults are suspected of having CAP (OR 3.4, 95% CI 1.1 to 10.0, p = 0.027); and always recommending an annual influenza vaccine to older patients (OR 4.2, 95% CI 2.6 to 6.7, p < 0.001)

**Table 3:** Strategies always or usually recommended by physicians to prevent pneumonia in their older adult patients.

	**Family Physicians**			**ER Physicians**			
		
**Prevention Strategy**	**Always %**	**Usually %**	***Combined %***	**Always %**	**Usually %**	***Combined %***	P-value*
Annual influenza vaccination	85.7	14.3	*100*	36.4	45.5	*81.9*	<0.05
Smoking cessation	84.1	15.9	*100*	54.5	36.4	*90.9*	>0.05
Pneumococcal vaccine	65.1	33.3	*98.4*	36.4	27.3	*63.7*	<0.01
Avoidance of tobacco smoke	48.4	35.5	*83.9*	36.4	54.5	*90.9*	>0.05
Frequent hand washing	20.6	44.4	*65.0*	18.2	18.2	*36.4*	>0.05
Nutritional programs	6.3	22.2	*28.5*	9.1	9.1	*18.2*	>0.05
Rehabilitation (OT and/or PT)	1.6	9.5	*11.1*	9.1	9.1	*18.2*	>0.05

*Comparison of "combined" percentages.

## Discussion

This survey provides a snapshot of how non-specialists, who care for the vast majority of older patients with CAP, practise in a typical non-urban Ontario community. The results provide insight into and highlight differences in approaches between office-based and emergency department physicians.

Although no individual element of the history and physical examination possesses a high enough likelihood ratio to establish a clinical diagnosis of CAP [10], one of the signs and symptoms that respondents reported seeing most frequently in patients with CAP (abnormal breath sounds) is associated with high positive likelihood ratios (up to 8.6) [11]. In contrast, shortness of breath, one of the most frequently reported signs and symptoms, has a positive likelihood ratio of only 1.4 [12]. These findings when considered in combination with self-reported levels of confidence in assessing CAP, suggest that there are clinical knowledge gaps that could be improved through research and education.

Differences in diagnostic testing between family and emergency department physicians were predictable. Although both groups ordered chest radiographs, the vast majority of ER physicians ordered complete blood counts and pulse oximetry compared to about only half of the family physicians. Since a greater proportion of patients with pneumonia seen by ER physicians will be admitted to hospital, this reflects the fact that ER physicians apply management standards for the hospitalized patient to a greater extent than family physicians. This is also a reflection of the increased severity of illness seen in patients presenting in the emergency department as well as the greater resources available in the ER compared to office practise. Whether a chest radiograph should be ordered for suspected pneumonia in the office setting is, however, an unanswered question. One study that randomly allocated chest radiographs to 1,500 consecutive patients with chronic cough, found a beneficial change in care to only 3% of patients [13]. Whether the same applies to patients with suspected pneumonia is unknown.

It is also notable that while the vast majority (92%) of ER physicians reported always using pulse oximetry, only 24% of family physicians reported (always or usually) using this technology. This again is likely a reflection of hospital management standards being applied in the ER, reflecting illness severity. Pulse oximetry is more readily available in emergency departments. However, pulse oximetry may be useful in the office setting, particularly when assessing older patients with chronic lung disease for pneumonia [14].

Some of the differences in attitudes and knowledge about CAP are a direct reflection of different clinical experience. For example, ER physicians would be expected to be more comfortable with their assessments of CAP given that they are more familiar with patients presenting with severe illness and they are more familiar with the management guidelines. Since age plays an important role in the Pneumonia Severity Index (3), this may be why more ER physicians were more likely to report age as being very important. Other differences relate to the amount of time spent with patients. For example, the lower rates of counseling for smoking cessation and immunization among ER physicians reflect the limited amount of time spent with each patient.

One of the most rigorous studies on CAP involved the derivation and validation of the Pneumonia Severity Index [3]. This index, created using an analysis based on over 14,000 patients and validated in a cohort of over 38,000, provides an accurate assessment of prognosis with respect to patients presenting with CAP. We found that the use of this index was reported relatively infrequently by family and ER physicians in our study. Our finding that fewer family physicians (12%) than ER physicians (50%) reported always or usually using the PSI is perhaps not surprising given the greater severity of illness typically seen in the ER. Although most physicians reported not using the index, one feature of the index that physicians did indicate was very important when assessing older patients with CAP was other co-morbidities (73% of respondents). Age, however, is one of the most important predictors of death, but overall only 26% of respondents felt that it was a very important characteristic to know when assessing/treating older adults suspected of having CAP.

We found that practice patterns among family physicians, such as requesting pulse oximetry, ordering chest x-rays for older patients, and using the Pneumonia Severity Index, were clustered. Although we did not have a large enough sample size to conduct a multivariable analysis, these physicians did state that CAP was an important concern for older patients in their practice. It may be that these physicians had a disproportionate number of older patients compared to other family physicians.

Regarding therapy, both family and ER physicians followed recommended Canadian guidelines for empiric therapy of CAP, with family physicians prescribing newer macrolides and emergency department physicians prescribing respiratory fluoroquinolones [15].

## Conclusion

The fact that the majority of family physicians (86%) reported always recommending influenza vaccine to patients compared to 36% in the emergency department raises the question as to whether the emergency department provides a good opportunity for immunization. The fact that physician practises tended to be clustered is also an interesting finding. For example, family physicians who ordered chest radiographs were more likely to order pulse oximetry, request follow up chest radiograph and to recommend pneumococcal vaccine.

The findings of this study provide a snapshot of how non-specialists from a non-urban Ontario community assess, manage and prevent CAP in older adults and highlight differences between office-based and emergency department physicians. Knowledge and beliefs about CAP were found to be associated with assessment, management and prevention strategies. An understanding of this connection between what physicians think and how they respond to CAP can guide researchers and clinicians in their ongoing efforts to improve the management and prevention of CAP in older adults.

## Competing interests

The author(s) declare that they have no competing interests

## Authors' contributions

PK had a major role in the conception and design of the study, supervised all aspects of the study's implementation, had a major role in data analysis and interpretation, and was the lead writer of this manuscript.

ML contributed to the conception and study design, participated in data analysis and interpretation, contributed to the writing of the manuscript, and provided editorial comments.

CK contributed to the implementation and acquisition of study data, participated in data analysis and interpretation, contributed to the writing of the manuscript and provided editorial comments.

GE contributed to the study design, implementation and acquisition of study data, critical review of the manuscript and provided editorial comments.

## Pre-publication history

The pre-publication history for this paper can be accessed here:


